# Knockdown of POLA2 increases gemcitabine resistance in lung cancer cells

**DOI:** 10.1186/s12864-016-3322-x

**Published:** 2016-12-22

**Authors:** Vivien Koh, Hsueh Yin Kwan, Woei Loon Tan, Tzia Liang Mah, Wei Peng Yong

**Affiliations:** 1 National University Cancer Institute Singapore, National University Health System, Singapore, Singapore; 2 Cancer Science Institute of Singapore, National University of Singapore, Singapore, Singapore; 3 Institute for Infocomm Research, Agency for Science, Technology and Research, Singapore, Singapore

**Keywords:** POLA2, Gemcitabine, Non-small cell lung cancer, Drug response, Single nucleotide polymorphism

## Abstract

**Background:**

Gemcitabine is used as a standard drug treatment for non-small cell lung cancer (NSCLC), but treatment responses vary among patients. Our previous studies demonstrated that POLA2 + 1747 GG/GA single nucleotide polymorphism (SNP) improves differential survivability and mortality in NSCLC patients. Here, we determined the association between POLA2 and gemcitabine treatment in human lung cancer cells.

**Results:**

Human PC9, H1299 and H1650 lung cancer cell lines were treated with 0.01-100 μM gemcitabine for 72 h. Although all 3 cell lines showed decreased cell viability upon gemcitabine treatment, H1299 was found to be the most sensitive to gemcitabine treatment. Next, sequencing was performed to determine if POLA2 + 1747 SNP might be involved in gemcitabine sensitivity. Data revealed that all 3 cell lines harbored the wild-type POLA2 + 1747 GG SNP, indicating that the POLA2 + 1747 SNP might not be responsible for gemcitabine sensitivity in the cell lines studied. Silencing of POLA2 gene in H1299 was then carried out by siRNA transfection, followed by gemcitabine treatment to determine the effect of POLA2 knockdown on chemosensitivity to gemcitabine. Results showed that H1299 exhibited increased resistance to gemcitabine after POLA2 knockdown, suggesting that POLA2 does not act alone and may cooperate with other interacting partners to cause gemcitabine resistance.

**Conclusions:**

Collectively, our findings showed that knockdown of POLA2 increases gemcitabine resistance in human lung cancer cells. We propose that POLA2 may play a role in gemcitabine sensitivity and can be used as a prognostic biomarker of patient outcome in NSCLC pathogenesis.

## Background

Lung cancer is a leading cause of cancer mortality worldwide, accounting for approximately 28% of all cancer deaths annually. The prognosis for lung cancer is poor. Most lung cancer patients are diagnosed at advanced stage and only 16% remained surviving 5 years after initial diagnosis. Out of all lung cancer cases, about 85–90% of diagnosis are non-small cell lung cancer (NSCLC) [1]. NSCLC consists of three major tumor subtypes: adenocarcinoma, large-cell carcinoma and squamous-cell carcinoma. Therapeutic regimens are platinum-based doublets, whereby the second agent can be microtubule-targeted agents, cytidine analogues or DNA-damaging agents [2]. Cytotoxic chemotherapy thus remains as the current standard cure for patients having advanced NSCLC.

Gemcitabine, a cytidine analogue, is well-known to have a significant therapeutic effect in NSCLC cases [3]. It is a prodrug that becomes activated by intracellular kinases to form di- and tri-phosphorylated metabolites, which together catalyze the conversion of ribonucleotides to deoxyribonucleotides and terminate DNA synthesis [4]. Studies have shown that the responses of NSCLC patients to gemcitabine treatment vary, which could be due to genetic polymorphisms and different gene variants involved in the gemcitabine pathway [5, 6].

The eukaryotic DNA polymerase α, one of the main polymerases involved in nuclear DNA replication, is a four-subunit (A, B, C, D) enzyme which possesses DNA polymerase and primase activities. Earlier biochemical studies have reported that subunit A displays catalytic activity, while subunits C and D exhibit primase activity [7, 8]. Subunit B, also known as DNA polymerase α2 accessory subunit (POLA2), is a 70-kDa regulatory subunit which contributes to DNA replication by binding the DNA polymerase α-primase complex to the initiation and elongation machinery [9]. POLA2 is widely expressed in a variety of tissue types. It has recently been shown to participate in gene fusion events and may also act as a prognostic biomarker in ovarian cancer and gastrointestinal stromal tumors [10, 11]. However, the exact role of POLA2 in human cancer remains unknown and its involvement in NSCLC pathogenesis remains understudied.

We have previously demonstrated that POLA2 + 1747 GG/GA improves differential survivability and mortality in NSCLC patients, and proposed that this novel single nucleotide polymorphism (SNP) may be used as a prognostic biomarker of patient outcome in NSCLC pathogenesis [12]. In the current study, we sought to determine the association between POLA2 and gemcitabine treatment, and further characterized the role of POLA2 in human lung cancer cells.

## Results

### Genomic landscape of POLA2

**Fig. 1:**
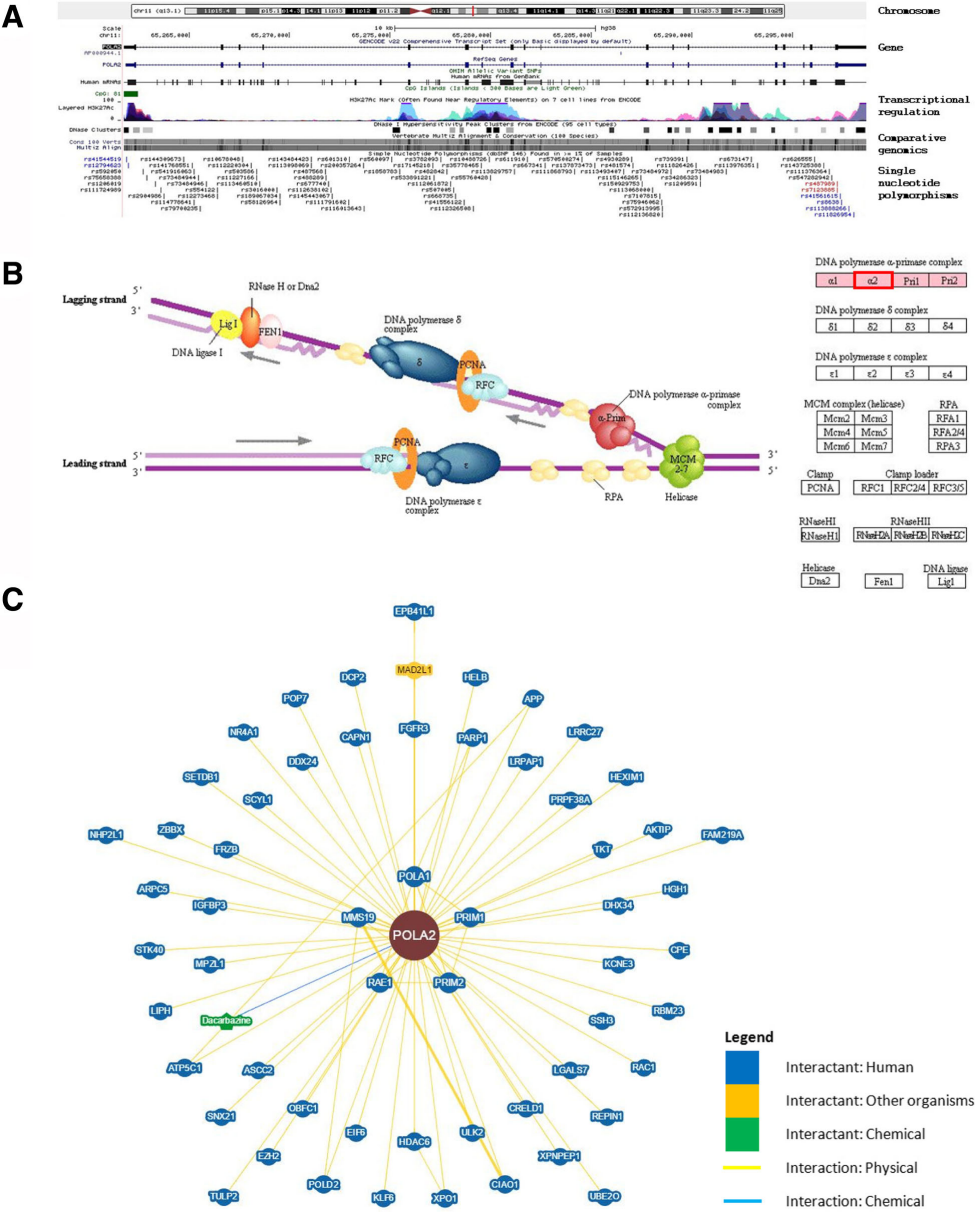
Genomics analysis of POLA2. **a** Genomic landscape of POLA2 based on the human Dec. 2013 (GRCh38/hg38) assembly. Location of POLA2 is indicated by a red vertical line on chromosome 11. Coding exons are represented by blocks linked by a horizontal line. CpG islands are shown as green blocks. Data associated with integrated transcriptional regulatory elements were retrieved from ENCODE. Comparative genomics analysis using multiple alignments of vertebrate species revealed conservation of the POLA2 gene. Each SNP is shown separately and labeled with the respective SNP ID reported by dbSNP build 146. Only SNPs that have a minor allele frequency of at least 1% and are mapped to a single location in the reference genome assembly are shown in the figure. **b** Eukaryotic DNA replication complex showing the role of POLA2 (boxed in red) in DNA replication, which involves a complex network of interacting enzymes and proteins. Three DNA polymerases (α, δ and ε) have been identified in eukaryotes. DNA polymerase α, including POLA2, forms a complex with DNA primase during the process. Source: Kyoto Encyclopedia of Genes and Genomes (KEGG) pathway database. **c** Network visualization of POLA2 and its interactants. Physical and genetic interactions, including chemical associations and post-translational modifications, were curated from published datasets and annotated interaction data from NCBI to construct this network

To understand POLA2 genomic sequence, we first examined the sequence of POLA2 using the human Dec. 2013 (GRCh38/hg38) assembly on the UCSC Genome Browser (https://genome.ucsc.edu/). POLA2 is located on chromosome 11q13.1 and contains 21 exons. Assessment of the DNA sequence using data retrieved from the Encyclopedia of DNA Elements (ENCODE) indicated the presence of several integrated transcriptional regulatory elements. Comparative genomics analysis using multiple alignments of vertebrate species revealed conservation of the POLA2 gene. We further explored the presence of SNP sites within the POLA2 genomic region. Figure 1a shows the location of each SNP, as reported by the Single Nucleotide Polymorphism database (dbSNP) build 146. Only SNPs that have a minor allele frequency of at least 1% and are mapped to a single location in the reference genome assembly are included.

### Association of POLA2 with other genes

Recent studies reported that POLA2 may participate in gene fusion events that can contribute to prognosis in ovarian cancer and gastrointestinal stromal tumors. Using data compiled from the National Center for Biotechnology Information (NCBI) and Gene Ontology (GO) framework databases, we uncovered 44 unique interactants for POLA2 (Table 1). These interactants were inferred from physical interactions, sequence or structural similarities, as well as direct experimental assays. Pathway analysis showed that POLA2 predominantly plays a major role in DNA replication, which involves a complex network of other interacting enzymes and proteins (Fig. 1b). Figure 1c further depicts the visualization of POLA2 and its interactants in a network, which was generated based on published and known physical or genetic interactions, chemical associations and post-translational modifications.

**Table 1:** POLA2 and its interactants based on published experimental evidence

GENE SYMBOL	GENE NAME	CHROMOSOMAL LOCATION	EXON COUNT	GENE ONTOLOGY (ACCESSION)			EXPERIMENTAL SOURCE
Gene of interest							
POLA2	Polymerase (DNA) alpha 2, accessory subunit	11q13.1	21	GO:0003887	GO:0006260		-
Interactants							
AKTIP	AKT interacting protein	16q12.2	11	GO:0001934	GO:0005515		Two-hybrid
APP	Amyloid beta precursor protein	21q21.3	20	GO:0045931	GO:0051425		Reconstituted Complex
ASCC2	Activating signal cointegrator 1 complex subunit 2	22q12.1	23	GO:0006355			Two-hybrid
ATP5C1	ATP synthase, H+ transporting, mitochondrial F1 complex, gamma polypeptide 1	10p15.1	10	GO:0006754	GO:0016887		Two-hybrid
CAPN1	Calpain 1	11q13	24	GO:0005515	GO:0008284		Affinity Capture-MS
CIAO1	Cytosolic iron-sulfur assembly component 1	2q11.2	7	GO:0005515	GO:0008284		Affinity Capture-MS
CPE	Carboxypeptidase E	4q32.3	9	GO:0050839	GO:0072657		Two-hybrid
CRELD1	Cysteine rich with EGF like domains 1	3p25.3	12	GO:0005509			Two-hybrid
DCP2	Decapping mRNA 2	5q22.2	11	GO:0005515	GO:0006402		Two-hybrid
DDX24	DEAD-box helicase 24	14q32	9	GO:0044822			Two-hybrid
DHX34	DEAH-box helicase 34	19q13.3	21	GO:0044822			Two-hybrid
EIF6	Eukaryotic translation initiation factor 6	20q12	8	GO:0005515			Two-hybrid
EPB41L1	Erythrocyte membrane protein band 4.1 like 1	20q11.2-q12	37	GO:0005515			Co-fractionation
EZH2	Enhancer of zeste 2 polycomb repressive complex 2 subunit	7q35-q36	25	GO:0005515	GO:0031490	GO:0042127	Two-hybrid
FGFR3	Fibroblast growth factor receptor 3	4p16.3	19	GO:0005515	GO:0008543	GO:0042127	Two-hybrid
FRZB	Frizzled-related protein	2q32.1	6	GO:0017147	GO:0043065		Two-hybrid
HDAC6	Histone deacetylase 6	Xp11.23	32	GO:0005515	GO:0016575	GO:0045892	Two-hybrid
HELB	Helicase (DNA) B	12q14.3; 12q	14	GO:0006260	GO:0044822		Reconstituted Complex
HEXIM1	Hexamethylene bis-acetamide inducible 1	17q21.31	1	GO:0005515	GO:0045892		Co-fractionation
IGFBP3	Insulin like growth factor binding protein 3	7p12.3	5	GO:0005515	GO:0043065		Two-hybrid
KCNE3	Potassium voltage-gated channel subfamily E regulatory subunit 3	11q13.4	3	GO:0005515	GO:0015459		Two-hybrid
KLF6	Kruppel-like factor 6	10p15	4	GO:0005515	GO:0045893		Two-hybrid
LRPAP1	LDL receptor related protein associated protein 1	4p16.3	9	GO:0070326			Two-hybrid
MAD2L1	MAD2 mitotic arrest deficient-like 1 (yeast)	4q27	5	GO:0005515	GO:0007093		Affinity Capture-MS
MMS19	MMS19 homolog, cytosolic iron-sulfur assembly component	10q24-q25	32	GO:0005515	GO:0045893		Affinity Capture-MS
NR4A1	Nuclear receptor subfamily 4 group A member 1	12q13	13	GO:0003677	GO:0005515	GO:0045786	Two-hybrid
PARP1	Poly(ADP-ribose) polymerase 1	1q41-q42	23	GO:0003677	GO:0005515	GO:0006281	Affinity Capture-Western; Far Western
POLA1	Polymerase (DNA) alpha 1, catalytic subunit	Xp22.1-p21.3	38	GO:0003887	GO:0006260		Co-fractionation; Reconstituted Complex
POLD2	Polymerase (DNA) delta 2, accessory subunit	7p13	13	GO:0003887	GO:0006260		Co-fractionation
POP7	POP7 homolog, ribonuclease P/MRP subunit	7q22	2	GO:0005515	GO:0044822		Co-fractionation
PRIM1	Primase (DNA) subunit 1	12q13	13	GO:0006270			Co-fractionation
PRIM2	Primase (DNA) subunit 2	6p12-p11.1	19	GO:0006270			Co-fractionation
PRPF38A	Pre-mRNA processing factor 38A	1p32.3	10	GO:0005515	GO:0044822		Two-hybrid
RAE1	Ribonucleic acid export 1	20q13.31	15	GO:0003723			Affinity Capture-MS
RBM23	RNA binding motif protein 23	14q11.2	19	GO:0003723	GO:0005515		Two-hybrid
REPIN1	Replication initiator 1	7q36.1	6	GO:0003677	GO:0006260	GO:0044822	Co-fractionation
SCYL1	SCY1 like pseudokinase 1	11q13	18	GO:0006355	GO:0006890		Co-fractionation
SETDB1	SET domain bifurcated 1	1q21	22	GO:0005515	GO:1990841		Two-hybrid
SNU13	SNU13 homolog, small nuclear ribonucleoprotein (U4/U6.U5)	22q13	5	GO:0005515	GO:0044822	GO:0051117	Two-hybrid
STK40	Serine/threonine kinase 40	1p34.3	12	GO:0010468	GO:0043066	GO:0043408	Two-hybrid
UBE2O	Ubiquitin conjugating enzyme E2 O	17q25.1	19	GO:0005515	GO:0006513	GO:0044822	Co-fractionation
ULK2	Unc-51 like autophagy activating kinase 2	17p11.2	29	GO:0005515	GO:0010506		Two-hybrid
XPNPEP1	X-prolyl aminopeptidase (aminopeptidase P) 1, soluble	10q25.3	23	GO:0042803			Two-hybrid
XPO1	Exportin 1	2p15	29	GO:0003723	GO:0005515	GO:0006611	Affinity Capture-MS

Gene symbol, gene name, chromosomal location, number of exons and Gene Ontology accession numbers of each gene are shown. A total of 44 interactants were found for POLA2, as inferred from physical interactions, sequence or structural similarities as well as direct experimental assays. Brief description of the experimental source is given. Data were compiled from the National Center for Biotechnology Information (NCBI) and Gene Ontology (GO) framework databases

### Effect of gemcitabine on human lung cancer cell lines

**Fig. 2:**
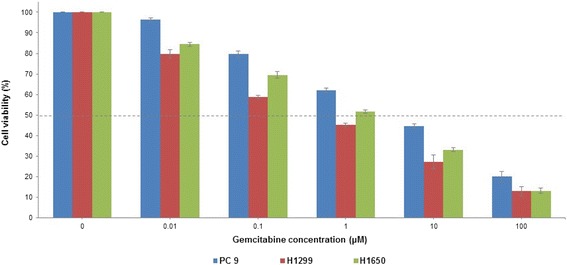
Effect of gemcitabine on human lung cancer cell lines. Human lung cancer cell lines, namely PC9, H1299 and H1650, were treated with 0.01–100 μM gemcitabine for up to 72 h. Untreated cells (0 μM gemcitabine) were used as the Control. All experiments were conducted in triplicates and repeated at least 3 times. Data are shown as the mean ± SD. Dotted line indicates 50% cell viability

To examine the effect of gemcitabine on human lung cancer cell lines, we treated PC9, H1299 and H1650 cell lines with 0.01–100 μM gemcitabine for 72 h. As shown in Fig. 2, cell viability decreased with increasing concentrations of gemcitabine treatment. Although all 3 cell lines showed similar trends of decreasing cell viability upon gemcitabine treatment, H1299 was found to be the most sensitive to gemcitabine, having the lowest IC_50_ among the 3 cell lines studied.

### Detection of SNPs in human lung cancer cell lines

**Fig. 3:**
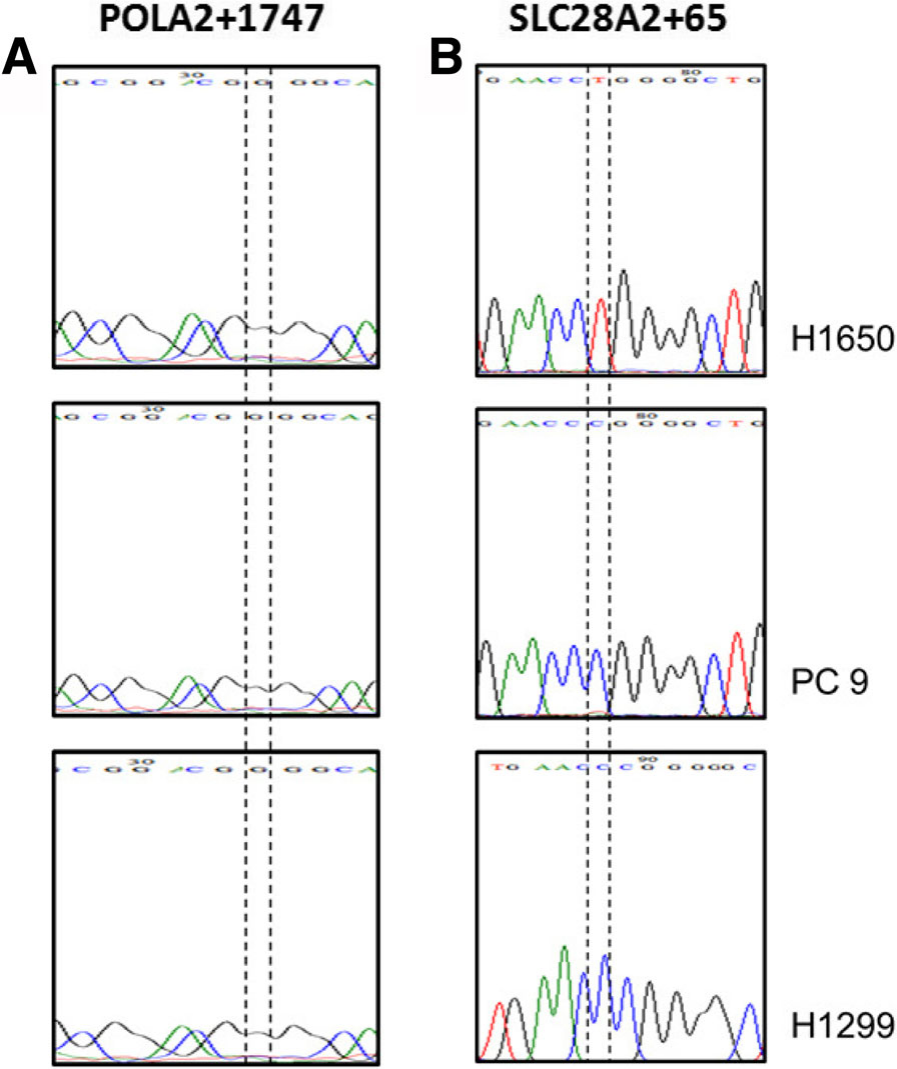
Detection of SNPs in human lung cancer cell lines. Sequencing plots of H1650, PC9 and H1299 cell lines for (**a**) POLA2 + 1747 SNP and (**b**) SLC28A + 65 SNP. All 3 cell lines harboured the wild-type POLA2 + 1747 GG SNP. Only H1650 harboured the mutation SLC28A + 65 CC > CT SNP, while both PC9 and H1299 contained the wild-type SLC28A + 65 CC SNP

The POLA + 1747 GG/GA SNP and SLC28A2 + 65 CC SNP are two of the 21 SNPs of the 9 genes involved in gemcitabine transport, metabolism and activity. From our in silico prediction analysis reported earlier [12], these two SNPs gave the best survival outcome and thus of interest for further studies in vitro. Here, sequencing of PC9, H1299 and H1650 cell lines revealed that all of these cell lines harboured the wild-type POLA2 + 1747 SNP (Fig. 3a), indicating that the POLA2 + 1747 SNP might not be responsible for gemcitabine sensitivity in the cell lines studied. Next, to investigate if SLC28A2 + 65 SNP might be involved in gemcitabine sensitivity, we sequenced PC9, H1299 and H1650 cell lines for the SLC28A2 + 65 SNP. As shown in Fig. 3b, only H1650 cell line harboured the mutation SLC28A2 + 65 CC > CT SNP, whereas both PC9 and H1299 cell lines contained the wild-type SLC28A2 + 65 CC SNP. We therefore selected H1299 and H1650 cell lines for subsequent experiments.

### Knockdown of POLA2 gene increased resistance to gemcitabine treatment

**Fig. 4:**
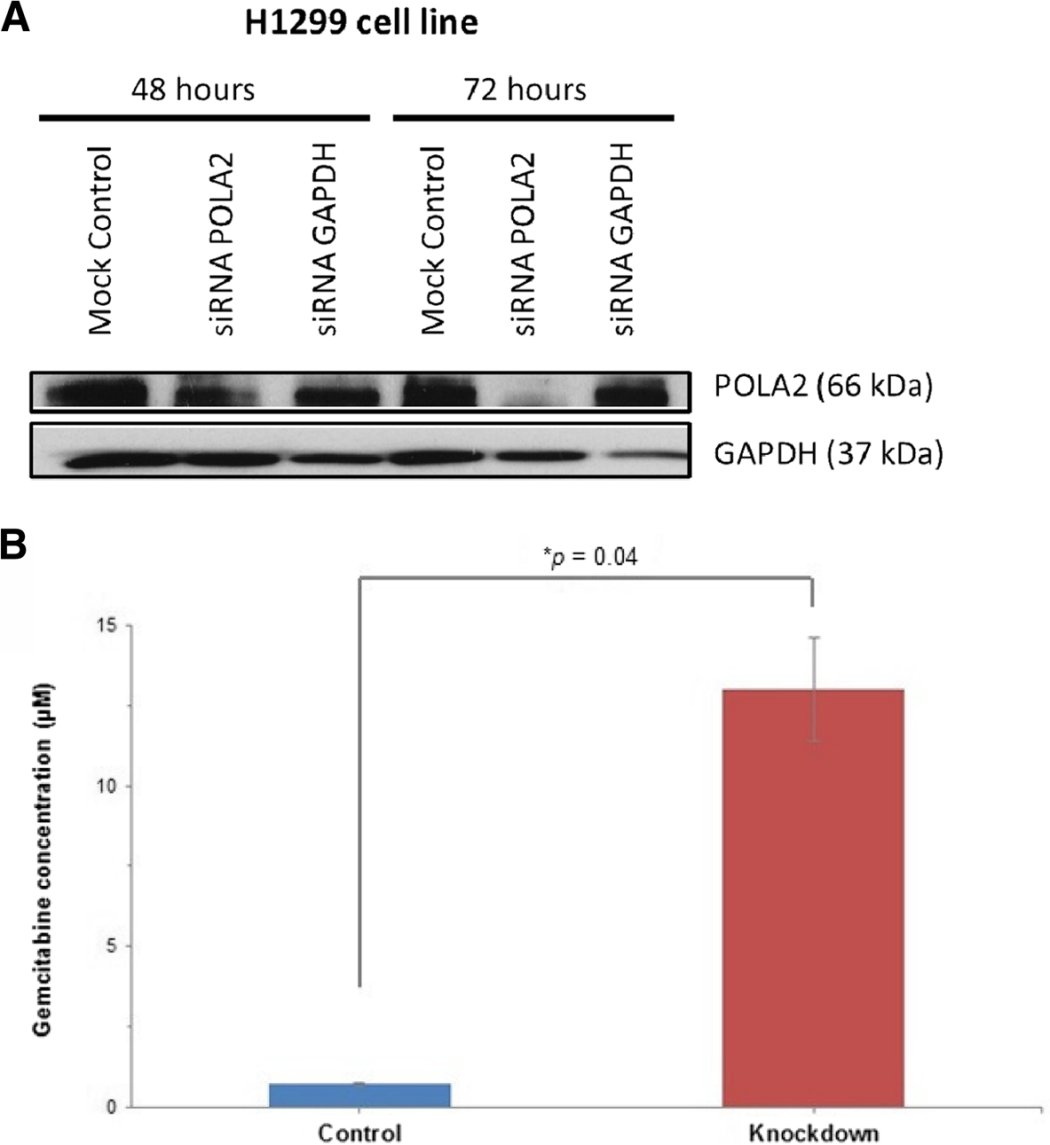
Effects of POLA2 gene silencing. **a** Protein expression after knockdown of POLA2 by siRNA. Comparison of POLA2 protein expression in H1299 cell line by Western blotting after 48 and 72 h of siRNA transfection. Internal control for equal loading: glyceraldeyde phosphate dehydrogenase (GAPDH). **b** Chemosensitivity assay by comparing IC_50_ values before and after knockdown of POLA2. Results indicated that the H1299 cell line became more resistant to gemcitabine treatment after siRNA knockdown of POLA2 (Knockdown), as compared to the non-transfected H1299 cell line having wild-type POLA2 (Control). Experiments were conducted in triplicates and repeated at least 3 times. Data are shown as the mean ± SD (*p <0.05)

Both H1299 and H1650 cell lines were transfected for 48–72 h with the respective small interfering RNAs (siRNAs) to silence POLA2 and SLC28A2 genes. However, H1650 cell line could not be successfully transfected (data not shown), hence only the H1299 cell line was used for further studies. Western blot analysis showed that POLA2 protein expression was significantly attenuated after 72 h of transfection (Fig. 4a). A chemosensitivity assay was then conducted to determine the effect of POLA2 knockdown on the drug sensitivity of H1299 cell line to gemcitabine. As shown in Fig. 4b, H1299 cell line became more resistant to gemcitabine upon knockdown of the POLA2 gene. This implied that other factors and mechanisms might play a role in contributing to gemcitabine resistance and more work needs to be done to explore this avenue.

## Discussion

Previously, we have demonstrated that the POLA2 + 1747 GG/GA SNP is associated with improved survivability and mortality in NSCLC patients [12]. In this current study, we reported that the response to gemcitabine treatment is related to POLA2 and confirmed that the knockdown of POLA2 gene increased the resistance to gemcitabine treatment in human lung cancer cells.

NSCLC is one of the most chemoresistant cancer types. Clinical treatment using traditional chemotherapy agents have reached a plateau in NSCLC patients due to resistance to chemotherapy drugs. Molecular targeted therapy through interruption of the epithelial growth factor receptor was one of the earlier treatment strategies used [13]. However, this strategy failed to achieve remarkable results during clinical trials [14]. Many factors, including genetic heterogeneity, redundant tumor growth as well as survival signaling pathways, likely contributed to the resistance of NSCLC patients to molecular therapies [15]. As such, genetic studies and the understanding of signaling mechanisms may help to identify appropriate treatment strategies for NSCLC patients.

The DNA polymerase α subunit B, which is involved in the initiation of chromosomal DNA replication, is encoded by the POLA2 gene. We have previously reported that different variants of the POLA2 gene may improve the prognosis of NSCLC patients [12]. We have demonstrated that the POLA2 + 1747 GG/GA SNP encodes for a mutant DNA polymerase α subunit B that is predominantly localized in the cytoplasm. This leads to an inhibition in nuclear DNA polymerase α activity, thereby conferring a protective effect on NSCLC patients due to termination of tumor DNA replication. This inhibition of tumor cell proliferation ultimately results in tumor cell death.

Gemcitabine is a potent DNA synthesis inhibitor, which is a deoxycytidine analogue with anti-tumor activity against various solid tumours such as NSCLC, pancreatic cancer, breast cancer and ovarian cancer [16]. Gemcitabine enters the cell through transport by members of the nucleoside transporter family SLC28 and SLC29 [17, 18]. It is then activated by the deoxycytidine kinase to its monophosphate form in a rate-limiting step. The monophosphate form is then further phosphorylated into gemcitabine triphosphate, which is then incorporated into the DNA by DNA polymerase α. Through a process of masked chain termination, DNA synthesis and repair are prematurely halted [19, 20]. As gemcitabine is involved in the inhibition of DNA synthesis, it is a suitable drug candidate for studying the role of POLA2.

One important aspect of genetic studies is the interactions between SNPs. In a study of SNP-SNP interactions, a small number of SNPs is genotyped and then tested if any interactions are present. Such SNP-SNP interaction studies may help to identify genomic hotspots in human diseases. The possibility of using statistical modeling by many types of regression techniques with straightforward implementation of interaction analysis is also an additional advantage of SNP-SNP interaction studies. We have applied biostatistics to calculate the probability between different SNP pairs and their overall association to the survival of lung cancer patients [12]. From in silico prediction using statistics, we found that POLA2 + 1747 GG/GA SNP together with SLC28A2 + 65 CC SNP were associated with increased median survival. Here, in this study, we validated these statistical findings through wet-lab experiments. We hypothesised that having the POLA + 1747 GG/GA SNP would cause a tumor to be more sensitive to gemcitabine treatment. By performing drug sensitivity tests and sequencing the DNA of human lung cancer cells for the SNP sites of interest, we were able to monitor whether the presence of POLA + 1747 GG/GA SNP could affect the response to gemcitabine treatment. Our results showed that all cell lines contained the wild-type POLA2 gene. We then proceeded to silence the POLA2 gene by siRNA transfection and found that instead of causing tumor cell death due to termination of DNA replication, the cell lines became more resistant to gemcitabine treatment. This is the first report to suggest that the POLA2 gene does not act alone but may cooperate with other genes to cause drug resistance to gemcitabine. Indeed, we showed through in silico prediction that several interacting partners of POLA2 exist. Our findings are concomitant with those recently reported by Kang et al*.* [10], who showed that POLA2 participates in gene fusion events and may act as a prognostic biomarker in gastrointestinal stromal tumors.

Earlier studies have reported other genes that caused resistance to gemcitabine treatment. Davidson et al*.* [21] proposed that increased expression of the ribonucleotide reductase catalytic subunit M1 (RRM1) gene results in increased resistance to gemcitabine, while Oguri et al*.* [22] reported that decreased expression of the multidrug resistance protein 5 (MRP5) gene leads to an increase in gemcitabine sensitivity. In addition, studies by Rha et al*.* [23] showed that patients with the RRM1 haplotypes 2455 A > G and 2464 G > A tend to be genetically more resistant to gemcitabine. Collectively, these studies showed that resistance to gemcitabine are multifactorial, which involves decreased intracellular accumulation and alteration of metabolism [24]. Similar factors include increased gemcitabine degradation enzymes, decreased gemcitabine regulation enzymes and decreased activity of nucleoside transporters (SLC28A1 and SLC29A1) [20, 25, 26]. Other factors such as activation of DNA repair pathways, negative regulation of apoptosis, alterations in cell cycle and cell proliferation pathways, and transition to a more epithelial-to-mesenchymal transition (EMT)-like phenotype can also lead to an increased resistance to gemcitabine treatment.

Our findings showed that POLA2 may be a novel gene that causes resistance to gemcitabine. Similar to the RRM1 gene, POLA2 is involved in DNA synthesis and hence, a decrease in its expression might cause the up-regulation of genes that play a role in DNA synthesis and repair. These genes and their polymorphisms should be studied to determine whether there are any SNP-SNP interactions present among them, as well as with the POLA2 gene, that could have led to gemcitabine resistance. Van de Wiel et al*.* [27] reported chromosomal aberrations as detected by microarray analysis. Genomewide gene expression and SNP microarray analyses may further reveal information on signaling mechanisms and pathways leading to acquired gemcitabine resistance. We are currently exploring this area to study which genes are deregulated, leading to drug resistance. Studying the gene expression and polymorphisms of nucleoside transporters and genes involved in DNA synthesis and repair can also help to predict chemosensitivity.

## Conclusions

Taken together, our findings suggested that POLA2 may play a role in gemcitabine resistance. This has important implications as patients with certain variants of the POLA2 gene may be resistant to gemcitabine and may not survive well on gemcitabine treatment. We propose that POLA2 may be used as a prognostic biomarker of patient outcome in NSCLC pathogenesis. More work needs to be done to characterize the correlation between POLA2 and gemcitabine resistance. Functions of the POLA2 + 1747 GG/GA SNP should also be further studied to determine its role in drug resistance and sensitivity. Our earlier report suggested that the presence of SLC28A2 + 65 CC/CT SNP may increase the sensitivity to gemcitabine and thus giving NSCLC patients a better prognosis [12]. However, as shown in Fig. 1 in our current study, SLC28A2 + 65 CC SNP has not been proven to be an interactant of POLA2. Therefore, more studies are needed to investigate whether SLC28A2 + 65 CC/CT SNP is associated with a better response to gemcitabine treatment and whether this SLC28A2 + 65 CC/CT SNP indeed interacts with the POLA2 + 1747 GG/GA SNP. A larger panel of cell lines carrying either or both SNPs needs to be screened to obtain a more comprehensive overview and we are currently working on this aspect.

## Methods

### Genomics analysis of POLA2

The genome sequence of POLA2 was examined using the human Dec. 2013 (GRCh38/hg38) assembly and Encyclopedia of DNA Elements (ENCODE) on the UCSC Genome Browser (https://genome.ucsc.edu/). Human POLA2 sequence was aligned with genome assemblies of other vertebrates and compared using Multiz Alignments Configuration. SNP sites within the POLA2 genomic region were investigated following the Single Nucleotide Polymorphism database (dbSNP; build 146). Only SNPs having a minor allele frequency of at least 1% and mapped to a single location in the human reference genome assembly were flagged and included in subsequent analyses.

### Study of SNP-SNP interactions

SNP-SNP interactions of 21 non-synonymous SNPs in 9 genes involved in gemcitabine transport, metabolism and activity were deduced in silico as reported previously [12]. Briefly, Fisher’s exact probability test was used to show that POLA + 1747 GG/GA SNP (rs487989) was the most statistically significant SNP to be associated with mortality. Chi-squared test was employed to assess the association between POLA + 1747 GG/GA SNP and SLC28A2 + 65 CC SNP, which confirmed that this SNP pair gave the best survival outcome [12]. All statistical tests were two-sided and performed using SPSS software version 14.0 (SPSS Inc., Chicago, IL). Differences were considered statistically significant when the *p*-value was less than 0.05.

### Association of POLA2 and other genes

Gene interactions in association with POLA2 were uncovered using data compiled from the National Center for Biotechnology Information (NCBI) and Gene Ontology (GO) framework databases. Interactants were inferred from physical interactions, sequence or structural similarities, as well as direct experimental assays. The Kyoto Encyclopedia of Genes and Genomes (KEGG) pathway database was used to further mine for major pathways involving POLA2. Only experimentally validated interactants were chosen to generate a network of interactions using the Biological General Repository for Interaction Datasets (BioGRID), in which POLA2 plays a central role.

### Cell culture

Human lung cancer H1650 (NCI-H1650) and H1299 (NCI-H1299) cell lines were purchased from American Type Culture Collection (ATCC; Rockville, MD). Human lung adenocarcinoma PC-9 cell line was purchased from RIKEN BioResource Center (Ibaraki, Japan). All cell lines were cultured in Roswell Park Memorial Institute (RPMI) 1640 medium (Gibco; Thermo Fisher Scientific, Waltham, MA) containing 10% fetal bovine serum (HyClone; GE Healthcare Life Sciences, Marlborough, MA) as recommended by the respective suppliers.

### Drug treatment

Cells were seeded into 96-well plates (Nunc; Thermo Fisher Scientific) at a density of 2 x 10^4^ cells per well in 100 μl culture medium and incubated at 37 °C overnight. Cells were treated with various concentrations of gemcitabine (Sigma-Aldrich, St. Louis, MI) on the following day to determine the IC_50_ values. Final concentrations of gemcitabine treatment were 0.01, 0.1, 1, 10 and 100 μM. Cell viability was determined using the CellTiter 96 AQueous One Solution Cell Proliferation Assay (MTS; Promega, Madison, WI) following manufacturer’s instructions.

### Sequencing

Cell lines were sequenced to confirm the presence of the POLA2 + 1747 and SLC28A2 + 65 SNPs (Axil Scientific, Singapore). Sequencing results were then correlated with the IC_50_ values to assess the SNP association previously reported [12].

### Silencing of POLA2 + 1747 by siRNA transfection

Silencing of the POLA2 gene was done by siRNA transfection using DharmaFECT transfection reagents (Dharmacon, Lafayette, CO) following manufacturer’s instructions. The H1299 cell line was used, as this cell line had been tested and validated by the manufacturer. Transfection durations were 24, 48 and 72 h. Western blotting was then performed to check for protein expression of the silenced gene, as described previously [12].
